# Association of early traumatic experiences of sexual abuse with well-being, self-esteem, resilience and close relationships during adulthood

**DOI:** 10.1192/j.eurpsy.2023.217

**Published:** 2023-07-19

**Authors:** A. Papadopoulou, E. Panopoulou, G. Kogolidou, K. Gkikas, P. Bali, E. Kaloudi, R. Gournellis, A. Douzenis, V. Efstathiou

**Affiliations:** 1 Second Department of Psychiatry, National and Kapodistrian University of Athens, “Attikon” University General Hospital; 2 Psychology Department, National and Kapodistrian University of Athens, Athens, Greece

## Abstract

**Introduction:**

There is evidence that early traumatic experiences may have a negative impact on critical components of psychosocial adjustment, while they may also adversely affect mental health during adulthood.

**Objectives:**

The aim this study was to investigate the association of early traumatic experiences and in particular sexual abuse with well-being, self-esteem, resilience and close relationships during adulthood.

**Methods:**

The study included 499 individuals (76.2% women), with a mean age of 24.2 years. Participants completed Early Trauma Inventory-Short form for early traumatic experiences’ assessment, Brief Resilience Scale for resilience evaluation, Mental Health Continuum-Short Form to assess well-being and Experiences in Close Relationships-Revised scale for adult romantic attachment assessment.

**Results:**

The majority of participants (98.2%) responded positively to at least one statement related to early traumatic experiences while 235 individuals (47.1%) reported that they had experienced sexual abuse. Furthermore, individuals who had experienced sexual abuse displayed lower levels of well-being (p<0.001), self-esteem (p<0.001) and resilience (p<0.001), but higher levels of anxious adult attachment (p<0.001) compared to individuals without such traumatic experiences.

**Conclusions:**

The findings of the present study highlight the importance of timely detection and holistic and integrated management of psychological needs of individuals who have experienced early traumatic experiences and especially sexual abuse.

**Disclosure of Interest:**

None Declared

